# Correspondence

**Published:** 1897-04

**Authors:** Olof Schwarzkopf

**Affiliations:** Chicago


### CORRESPONDENCE.

---

EDITORS JOURNAL OF COMPARATIVE MEDICINE, ETC.

DEAR SIRS: In the February issue of your JOURNAL is a communication by Mr. Harold Sorby, Chicago, manager of the Pasteur Vaccine Company, in which he announces the discovery that "equine anasarca" is due to a variety of the streptococcus, and that it can be successfully treated by the antistreptococcus serum (Marmorek).

As it appeared to me questionable that anasarca, as understood in its general pathological sense, should be a germ disease, I procured a copy of the *Recueil de médecine vétérinaire*, containing the original announcement. This had been made at a regular meeting of the Central Society for Veterinary Medicine of Paris, and from the tenor of the discussion by Dr. Trasbot and others it is evident that the French veterinarians understand by "anasarca vrai" the disease known to English veterinarians as "purpura hemorrhagica."

In this version the discovery becomes at once intelligible, and is a verification of an idea which many of us have held for some time. Moreover, it is very interesting and important news for veterinarians.

OLOF SCHWARZKOPF.

CHICAGO, March 6, 1897.

